# Advances in the molecular mechanisms of zinc-finger transcription factors in neurodevelopmental disorders

**DOI:** 10.1016/j.ibneur.2025.02.010

**Published:** 2025-02-26

**Authors:** Hailin Wang, Ying Yang, Ziwei Ni, Xiaoting Qiao, Yaqian Guo, Xiaomin Wang, Duo Cao, Yayun Wang, Cailian Ruan

**Affiliations:** a Medical School of Yan’an University, Yan'an, Shaanxi 716000, China; b Shaanxi Institute for Pediatric Diseases, Xi'an Children's Hospital, Xi'an, China; c Xi'an Children's Hospital Research Institute, Xi'an, Shaanxi 710003, China; d School of Life Sciences, Yan'an University, Yan'an, Shaanxi 716000, China; e National Experimental Center of Air Force Medical University, Xi'an, Shaanxi 716000, China; f Xi'an Jiaotong University School of Medicine, Xi'an, Shaanxi 710032, China

**Keywords:** Neurodevelopmental disorders, Zinc-finger transcription factors, *BCL11A*, *BCL11B*

## Abstract

Neurodevelopmental disorders (NDDs) constitute a heterogeneous group of early-onset brain dysfunction disorders, which may arise from genetic or acquired etiologies. These disorders are characterized by behavioral and cognitive deficits that predominantly manifest during childhood development, thereby potentially impairing an individual's performance in learning, sports, and social situations. A comprehensive understanding of the pathogenesis of NDDs is crucial for the development of targeted therapeutic interventions. Zinc-finger transcription factors (ZFPs) play a pivotal role in regulating gene expression by modulating the binding of RNA polymerase to DNA, thereby either activating or repressing gene transcription. In recent years, the *BCL11* gene family of ZFPs has garnered significant attention due to its critical involvement in nervous system development. This review aims to elucidate the structure and molecular functions of the *BCL11* gene family, discuss its impact on the development of the central nervous system, and explore its association with neurodevelopmental disorders.

## Introduction

**Fig. 1:**
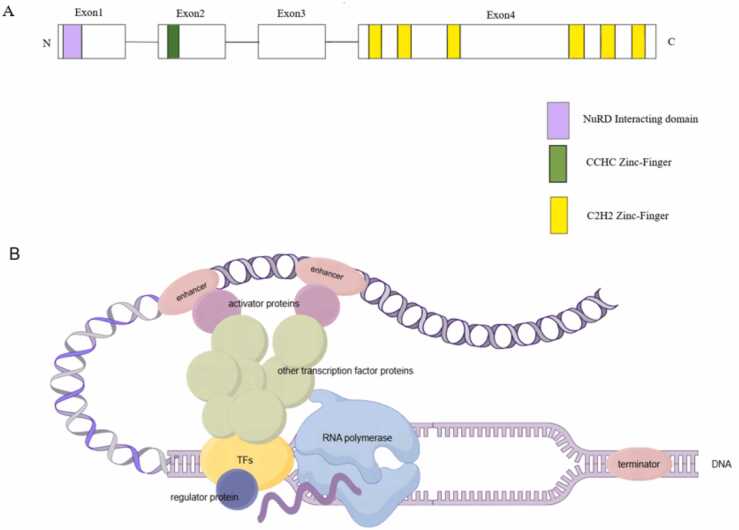
Bcl11a-mediated NDDs and the function of Bcl11a in central nervous system development. (A) The structure of isoforms of zinc-finger transcription factors BCL11A and BCL11B. The zinc-finger transcription factor BCL11 includes the NuRD interacting domain on exon 1, the CCHC Zinc-Finger on exon 2, and the C2H2 Zinc-Fingers on exon 4.

Neurodevelopmental disorders (NDDs) represent a category of brain dysfunction disorders that arise from a complex interplay of intrinsic genetic factors and extrinsic factors such as environmental influences and infections. These disorders include Autism Spectrum Disorder (ASD), Developmental Delay, Intellectual Disability (ID) and others (Thapar et al., 2017). These disorders share common clinical manifestations and exert varying degrees of mental, physical, and economic burdens on affected individuals, their families, and society at large (Seigfried and Britsch, 2024). Early identification and intervention of NDDs are crucial, as they can substantially enhance the quality of life for those affected (Al-Naama et al., 2020). Recently, two zinc-finger transcription factors, BCL11A and BCL11B, have been reported to be involved in the development of NDDs. Transcription factors (TFs) are instrumental in driving gene transcription by binding to specific regulatory elements within the genome, playing a pivotal role in brain development and the maintenance of its functions (Santiago and Bashaw, 2014). Disruptions in TFs, such as mutations, deletions, or other alterations, can lead to a spectrum of diseases, including ASD, Alzheimer's disease, and Schizophrenia (Simon et al., 2020). TFs are composed of a DNA-binding structural domain, a transcription activation structural domain, and a regulatory protein-binding regulatory structural domain (Santos-Terra et al., 2021). Among these, those containing zinc-finger domains are classified as zinc-finger transcription factors (ZFPs). ZFPs are a specialized class of TFs that contain one or more zinc-finger domains that allow them to bind specifically to DNA sequences, thus enabling them to precisely regulate gene expression and constitute the diversity and complexity of ZFPs (Laity et al., 2001). Research has identified associations between mutations in the *BCL11* gene family and various NDDs, and the interaction of *BCL11* gene family with other different cofactors will have an impact on neurodevelopment. These findings offer valuable insights into the role of the *BCL11* gene family in neurodevelopment and provide a theoretical foundation for the development of targeted, specific therapeutic approaches. Fig. 1

## Structure of zinc-finger transcription factor BCL11 and its function in nervous system development

The BCL11 zinc-finger transcription factors were initially identified through yeast two-hybrid screening as binding partner of the nuclear hormone receptors COUP-TF family. They can directly and specifically interact with all members of the COUP-TF family, thereby inhibiting the expression of target genes (Avram et al., 2000). The *BCL11* genes are highly conserved Krüppel-like C2H2 zinc-finger protein transcription factors (Durum, 2003), their homology in humans and mice is 95 % (Huang et al., 2012). The *BCL11* gene family consists of *BCL11A* and *BCL11B*, (Simon et al., 2020) they have 61 % identity at the protein level, 67 % identity at the nucleotide level, and 95 % identity at the zinc-finger domains (Avram et al., 2002), which indicates that they may have similar biological functions. BCL11A consists of 835 amino acids and BCL11B consists of 894 amino acids. These amino acids can form multiple C2H2-type zinc-finger domains in a specific order. Each zinc finger is composed of a β-sheet and an α-helix, stabilized by zinc ions and a series of hydrophobic residues The spatial folding of the entire protein chain results in a compact three-dimensional structure for BCL11, with the correct folding and arrangement of the zinc-finger domain being crucial for its function (Viennet et al., 2024). The *BCL11* gene family contains a nucleosome remodeling and deacetylase (NuRD) interacting domain, a CCHC zinc-finger domain and six C2H2 zinc-finger domains (Liu et al., 2006). The cluster of three C2H2 zinc-finger domains at the C-terminus enables transcriptional regulation of target genes by binding to specific DNA sequences, in which Bcl11a recognizes GGCCGGAGG and Bcl11b recognizes GGCCG/AG/AGG (Avram et al., 2002). Wakabayashi et al. (2003) demonstrated that in mice, the absence of this domain in *Bcl11b* leads to perinatal death of the embryo. The N-terminal CCHC zinc-finger domain mediates Bcl11 dimerization and nuclear translocation, and is intimately linked to transcriptional regulatory activity (Grabarczyk et al., 2018). The NuRD complex can interact with Bcl11 via the NuRD interacting domain, thereby coupling chromatin remodeling functions to histone modifications (Hoffmann and Spengler, 2019).

BCL11 is a lineage-specific transcription factor that can be expressed in a wide range of cells, mainly in the hematopoietic system and the central nervous system (CNS) (Kuo et al., 2010). In the human brain, *BCL11* family genes are primarily expressed at the RNA level in the Cerebral cortex, Hippocampus, Amygdala, Basal ganglia, Thalamus, Hypothalamus, Midbrain, Cerebellum, Pons, Medulla oblongata, Spinal cord, White matter and Choroid plexus. At the protein level, BCL11A is mainly expressed in the Cerebral cortex, Hippocampus, Caudate nucleus and Cerebellum. BCL11B is mainly expressed in the Cerebral cortex, Hippocampus and Caudate nucleus. The temporal expression of *BCL11A* in the CNS is largely concentrated during the embryonic development stage. *BCL11A* is highly expressed in prenatal-specific brain regions, including the Cerebral cortex, hippocampus and cerebellar cortex (Leid et al., 2004a). Shortly before birth, the expression levels of *BCL11A* in the Cerebral cortex, cerebellar cortex, hippocampus, amygdala, and mediolateral nucleus of the thalamus decreased rapidly (Bruce and Peter, 2022a).

In mice, Xuzhen et al. utilized Western blot and qPCR to verify the high-expression time point of Bcl11a in the mouse cortex. They found that the expression of Bcl11a in embryonic brain of E11.5 days, E13.5 days, E15.5 days and E17.5 days was higher, and the expression of Bcl11a in E13.5 days was the highest. In adult mice, Bcl11a is mainly expressed in the neocortex, hippocampus and striatum. Leid et al. (2004b) detected the expression of Bcl11b across different developmental stages in mice, and found that Bcl11b was diffusely expressed at E10.5 in mouse embryos, began to concentrate in the cerebral cortex, limbic system, basal ganglia and hippocampus at E12.5, and exhibited higher expression levels in the spinal cord at E14.5. In adult mice, Bcl11b is expressed in the cortex, hippocampus and basal ganglia, and plays a crucial role in adult neurogenesis in the dentate gyrus. Specifically, it primarily affects the specification, maintenance, and neuronal integration of newly born adult hippocampal granule cells.

In the human brain, BCL11A is expressed in the cerebellum, whereas BCL11B is absent in this region. This differential expression pattern suggests that these two transcription factors possess distinct functions within the nervous system. BCL11A likely plays a key role in the development and function of the cerebellum, while BCL11B mainly plays an important role in the function of other brain regions. This divergence reflects their unique pathological mechanisms in various neurodevelopmental disorders. The distinct spatiotemporal expression profiles of Bcl11a and Bcl11b underscore their specific roles at different developmental stages and their impact on neurodevelopment and the maintenance of neural function.

## *BCL11A*-mediated NDDs and the function of BCL11A in central nervous system development

In humans, the *BCL11A* gene locus is associated with Dias-Logan syndrome (OMIM: 617101), which is characterized by delayed psychomotor development, impaired intellectual development, variable dysmorphic features, including microcephaly, downslanting palpebral fissures, strabismus, and external ear abnormalities (Bruce and Peter, 2022b). Additionally, individuals with this syndrome exhibit asymptomatic persistence of fetal hemoglobin, ASD, childhood apraxia of speech and autonomic dysfunction (Peron et al., 2024). Patients with Bcl11a truncation mutations exhibit symptoms such as neurodevelopmental delays, severe language deficits, attention deficits, and enlarged ventricles (Bruce and Peter, 2022a). Magnetic resonance imaging (MRI) studies have revealed that the most common structural brain damage associated with this syndrome occurs in the cerebellum, with cerebellar vermis hypoplasia being a frequent finding (Peron et al., 2024; Shimbo et al., 2017). These findings highlight the critical role of Bcl11a in neurodevelopment and the potential impact of its mutations on brain structure and function.

Bcl11a is implicated in the migration, differentiation and survival of cortical neurons, as well as in axon formation (Seigfried and Britsch, 2024). Neurons deficient in Bcl11a are unable to shift from multipolar to bipolar morphology and exhibit delayed neuronal migration (Wiegreffe et al., 2015).Moreover, Bcl11a knockdown results in increased axon branching, dendritic growth and multiple axon formation, as well as the inhibition of active histone modifications at the promoter sites of neuron-associated factors (Kuo et al., 2009a). In the CNS, Frzb serves as a functional downstream target of Bcl11a and is involved in the differentiation of dorsal spinal cord neurons and the formation of sensory pathways, a function that is mediated primarily through the role of Bcl11a in the transcriptional control of Frzb (John et al., 2012). Bcl11a binds to the promoter region of Frzb, thereby regulating the transcription of Frzb, and the expression of Frzb is significantly down-regulated in Bcl11a mutant mice. John et al. (2012) conducted microarray analysis of embryonic dorsal spinal cord tissue from Bcl11a mutant mice and found that Frzb was downregulated in the dorsal spinal cord of these mice, leading to innervation defects in sensory neurons. Similarly, Frzb mutant mice also showed similar defects, indicating that the downregulation of Frzb expression is part of the reason for the innervation defect of Bcl11a mutant mice. Consequently, BCL11A modulates the Wnt signaling pathway by directly regulating Frzb expression, thereby influencing the axon guidance of sensory neurons and the formation of neural circuits. Another factor that interacts with Bcl11a to regulate gene expression is the BAF complex (Simon et al., 2020). The BAF complex regulates the transcriptional activity of genes by altering chromatin structure to allow transcription factors to access specific gene promoter regions (Varga et al., 2021). TBR1, a neuron-specific transcription factor highly expressed in the deep cortex, is another target gene of Bcl11a (Bedogni et al., 2010). Patients with TBR1 mutations present symptoms similar to those associated with Bcl11a mutations, including ID, ASD, and speech language problems (den Hoed et al., 2018). The C-terminal region of TBR1 and the amino acid 629–773 region of Bcl11a effectively bind Bcl11a to TBR1 in the cortex (Cánovas et al., 2015; Sabbagh et al., 2024). Through protein-protein interactions, they regulate gene networks crucial for neurodevelopment, encompassing the differentiation of cortical neurons and synapse formation, thereby impacting neurodevelopment.

## *BCL11B*-mediated NDDs and the function of BCL11B in central nervous system development

In humans, the *BCL11B* gene locus is associated with intellectual developmental disorder with speech delay, dysmorphic facies, and T-cell abnormalities (IDDSFTA) (OMIM:618029) and severe combined immunodeficiency-49 (IMD49) (OMIM:617237). Individuals with IDDSFTA commonly present with symptoms of ID, language delay, behavioral abnormalities, spatial learning and impaired memory (Sabbagh et al., 2024). IDDSFTA and IMD49 share overlapping features, including ID. Mutations in Bcl11b encompass frameshift, missense, nonsense mutations and chromosomal rearrangements (Seigfried and Britsch, 2024), with truncating mutations being predominant in neurodevelopmental disorders (García-Aznar et al., 2024). These mutations not only disrupt the binding of Bcl11b to target DNA sites but also promote its binding to new DNA sites (Lessel et al., 2018). The severity of these effects is largely contingent upon the location of the mutation.

Bcl11b plays a critical role in the differentiation and development of various neuronal subtypes within the CNS. Its function in the hippocampus has been comprehensively investigated, with Bcl11b being expressed in this region throughout both development and adulthood (Simon et al., 2016). In the hippocampus, Bcl11b regulates the proliferation, differentiation, maturation, and functional integration of progenitor cells, as well as synapse and axon formation (Simon et al., 2012). In the striatum, Bcl11b is essential not only for promoting the differentiation of medium spiny neurons but also for their functional maturation, thereby playing a key role in motor control (Arlotta et al., 2008). Deletion of Bcl11b results in damage to corticospinal motor neurons, a decreases in the number of synapses, reduces axon growth and pruning, and affects the proliferation of progenitor cells. Among these changes, axonal and synaptic alterations leading to impaired signaling function are the primary determinants of NDDs (De Bruyckere et al., 2018). Bcl11b is required for the establishment and maintenance of neuronal connectivity and has been implicated in the etiology of NDDs (Seigfried and Britsch, 2024). Bcl11b can bind to different cofactors and exert diverse functions. Acharawan et al (Topark-Ngarm et al., 2006). conducted immunoprecipitation experiments in SK-N-MC Cells and found that Bcl11b stably binds to and interacts with the NuRD complex. In a neuron-like environment, the NuRD complex plays a role in Bcl11b-mediated transcriptional repression (Kumar and Wang, 2016). The NuRD complex is an ATP-dependent chromatin remodeling complex with histone deacetylase activity (Lai and Wade, 2011), likely conferred by nucleosome remodeling and deacetylation. The MTA1 protein, a component of the NuRD complex, is involved in this process. Bcl11b recruits MTA1 to the target promoter and interacts with it through the amino-terminal region, and overexpression of MTA1 enhancing transcriptional repression (Cismasiu et al., 2005). The transcription factor Satb2, which is expressed in the upper neurons of the cerebral cortex and is essential for establishing connections between neurons in the inner cortex layers, represses Bcl11b expression by recruiting the NuRD complex to the NuRD-interacting domain of Bcl11b, thereby blocking transcription (Leone et al., 2015). Additionally, Bcl11b alters chromatin structure and accessibility through the action of scaffolding proteins (Wakabayashi et al., 2003), which can cause damage in the hippocampus and motor neurons.

## Conclusions

BCL11A and BCL11B are co-expressed only in the early cortex (Simon et al., 2020). BCL11 transcription factors binds to specific DNA through their C-terminal zinc-finger domains to regulate transcription, exhibiting a high affinity for TGACCA sequences (Liu et al., 2018). Through selective splicing, BCL11A produces four isoforms (BCL11A-XS, BCL11A-S, BCL11A-XL, BCL11A-L), and BCL11B produces two isoforms (Alpha, Beta). These isoforms are expressed in different tissues to varying extents, but there is no specific function for individual isoforms in the brain (Simon et al., 2020). BCL11A-XL and BCL11A-L are two long BCL11A isoforms, which play an important role in DNA interaction and localization to the nucleus. The short isoforms,BCL11A-S and BCL11A-XS, lack DNA-binding zinc-finger domains and are dominant in the cytoplasm. BCL11A-XL, along with BCL11A-S and BCL11A-L, can inhibit transcription. Specifically,BCL11A-L can regulate the direction and branching level of axon outgrowth (Kuo et al., 2009b). BCL11A-S can form heterodimers with the long isoforms, which can be transferred to the nucleus (Seigfried and Britsch, 2024; Liu et al., 2006). Haploinsufficiency of *BCL11A* and *BCL11B* has been implicated in the development of NDDs, including ID, developmental speech delay, motor delay, and ASD. Although the BCL11 gene family is known to be involved in central nervous system development and has been associated with the onset and progression of NDDs, the precise sites and molecular mechanisms of BCL11's involvement in NDDs remain to be elucidated. Therefore, extensive research is warranted to explore the specific pathological mechanisms and develop targeted therapeutic approaches. Technological advancements, such as the creation of the Hyper-Kvasir dataset, have demonstrated potential in enhancing diagnostic accuracy and supporting clinical decision-making, thereby improving disease diagnosis (Yang et al., 2024a). Meanwhile, the online databases and cases are used to collect patient information, which can be analyzed using the Retrieval-Augmented Generation(RAG) software system, which can track the changes in patients' conditions and their recovery (Yang et al., 2024b), providing the necessary technical support for the early diagnosis, intervention, and treatment of NDDs. For instance, constructing research models of NDDs through induced pluripotent stem cell technology to support the subsequent understanding of the pathogenesis of NDDs. Additional, the establishment of animal models is of paramount importance for investigating the pathogenic mechanisms and therapeutic strategies of NDDs. Yamaguchi et al. (2023) utilized a Drosophila model to investigate the effects of knocking down the Cph gene, a homolog of Bcl11, on the nervous system. They found that nervous system-specific knockdown of Cph resulted in larval learning behavior and motor deficits as well as epileptic-like behavior in adults, which closely resembled the pathological phenotypes of NDDs. This suggests that neuron-specific Cph knockdown in Drosophila may serve as a valuable model for NDD research and can be utilized to study the functions of specific genes. Wiegreffe et al. (2023) proposed the construction of a mouse model using the i-GONAD method, which exhibits symptoms akin to those observed in patients with BCL11 mutations. This model is instrumental for analyzing the function of TFs in specific cells and is crucial for understanding the role of the *BCL11* gene family in neurological development and related diseases.

In future experiments, we aim to establish NDD research models through induced pluripotent stem cell differentiation and construct mouse models to investigate their pathogenesis, with the goal of providing improved treatment options for patients.

## Ethical statement

All animal experiments comply with the ARRIVE guidelines.

## Consent for publication

All the authors gave their consent for the article to be published in this journal.

## Funding

This work was supported by the 10.13039/501100001809National Natural Science Foundation of China (82201627), the Military Medicine Upgrade Program of Air Force Military Medical University (2020SWAQ04), Shaanxi Provincial Innovation Capacity Support Programme (2023-CX-PT-33), Shaanxi Provincial Natural Science Basic Research Programme (2024JCZDXM-60, 2022JQ-820), Shaanxi Provincial Natural Science Basic Research Programme Key Projects (2024JC-ZDXM-51), and Xijing Hospital Clinical New Technology (2023XJSY27).

## Authors’ contributions

All authors contributed to the study conception and design. The authors thank CailianRuan for competing the trials and figure design. The authors thank HailinWang and YingYang for critical comments on the manuscript. All authors read and approved the final manuscript.

## Data and materials availability

This article did not look at any new data. Only results published in previous studies and identified in the reference list below were used.

## Declaration of Competing Interest

The authors declare that they have no competing interests.
